# Annotations

**Published:** 1909-11-06

**Authors:** 


### Annotations.

### The Wealth of Consultants.

There is a prevailing impression among the laity that the average successful consultant is a man of means, a roller in wealth, and an amasser of vast fortunes. We who are in the profession know how little truth there is in these vague statements to the effect that So-and-So is making so many thousands a year, but the public at large is scarcely in a position to judge of the reliability of these exaggerations. This week, however, it has the opportunity of noticing how small these fortunes are. Dr. Clapton, consulting physician to St. Thomas's Hospital, after more than half a century of active work, leaves some £43,000, while Sir Stephen Mackenzie, consulting physician to the London Hospital, after a length of service almost equally long, leaves about a fourth of that sum. These consultants, during their period of active service, stood very high in the profession, and when one compares their fortunes with those left by prominent lawyers, stockbrokers, or merchants, it is evident that they must have worked—and worked hard—at a much lower valuation than members of the other professions or trades.

### Veronal.

The inquest held this week on a gentleman who succumbed to what was evidently an overdose of veronal, and in which the jury thought fit to add a rider to their verdict to the effect that some restrictions should be placed on the sale of the drug, draws attention to the danger of the indiscriminate use of veronal in particular and synthetic "sleeping drugs" in general. Veronal, or to give it its full scientific name, Diethylbarbituric acid, has now been in use for more than ten years, and, in the practitioner's hands, it has proved one of the safest and least objectionable of hypnotics or anodynes. It is given in comparatively large doses, and with fair success, as a prophylactic against sea sickness, while as a pure hypnotic it is certainly both efficient and rapid. At the same time, it is by no means a drug that ought to be sold without a definite prescription, or that ought to be taken by patients not under medical supervision, and some restriction is the more indicated since there is reason to believe that some people possess a definite idiosyncratic intolerance to veronal. As a habitual hypnotic, especially, its use ought to be regulated by the medical attendant, and there is no excuse for permitting patients to take it when they please and how they please.

### The Bermondsey Election.

The by-election recently concluded has had its semi-tragic and its ultra humorous side. As a matter of fact, it has been an election bristling with points of interest for the medical man. For the first time in Parliamentary history a member of the medical profession—a profession which, as is well known, is curiously under-represented in the House of Commons—standing as an avowed Socialist, has solicited the support of an electorate. That he has been unsuccessful is doubtless due to a variety of reasons, into which we need not go, for the majority of them are political and, therefore, polemical. Medical men have a right to individual opinions on matters political, and, to some degree—let us hope a very small degree—Dr. Salter's professional colleagues opposed him because he held socialistic opinions, and not because they did not fully realise the desirability of having so brilliant a member of the profession in Parliament. The humorous side of the struggle was seen largely in the frantic attempts of the antivivisectionists to make capital out of the medical candidate's common-sense replies, and in the egregious letter with which the residents of Guy's Hospital thought fit to favour the Press. Much may be forgiven the militant "suffragette" or the vigorous Hadwenite, but we confess to a mild surprise that a number of young men, who are presumably not altogether devoid of a sense of humour, should have had the courage to imagine that the public cared for a moment to know what the residents of Guy's Hospital thought about Dr. Salter's candidature.

### Leprosy.

The delegates of the British Government to the International Congress on Leprosy, which met at Bergen in August last, have just issued their official report, which is published in the form of a Parliamentary white paper (Cd. 4916). The delegates give details of the various resolutions passed by the Congress, to which they agreed. The most important of these resolutions affirm the contagious theory of leprosy, urge the strict isolation of all sufferers of the disease, the desirability of isolating the children of leprous parents, and the importance of research work into the ætiology and causation of leprosy generally. With reference to the last resolution, it is interesting to note that the Congress, while affirming that all theories with regard to the ætiology of the disease are worth investigating, lays particular stress on the desirability of devoting attention to the elucidation of the question of transmissibility by insects, and to the importance of instituting a strict inquiry to find out whether or not leprosy or leproid disease affects animals such as rats. Finally, the Congress arrived at the conclusion that while there is no specific remedy for the disease, leprosy is not incurable, and that it is necessary to investigate carefully the claims of the various drugs which are lauded in the treatment. On the other hand, the Congress decided that fish-eating has nothing to do with the disease and that there is no evidence to warrant a belief that it is hereditary. As a mere outline of the present state of the case for and against leprosy, the report is interesting, though it adds nothing to our knowledge of the disease and the resolutions cited are open to discussion. The main point is that the report will draw attention to the urgent necessity for investigating the disease. While in England leprosy is a very rare affection, it must always be borne in mind that in some of our colonies it is an ever-present problem which demands the closest consideration in the interests of the community.

